# Intraventricular hemorrhage, suspected EBV reactivation, and TBA-positive epilepsy after deep cervical lymphovenous anastomosis in Alzheimer’s disease: a case report

**DOI:** 10.3389/fnagi.2026.1791011

**Published:** 2026-04-01

**Authors:** Tiantian Jiang, Fang Yan, Bin Liu, Qiuju Li, Kan Wang, Xinyi Ru, Yong Hao, Yangtai Guan, Yuhui Wang

**Affiliations:** 1 Department of Neurology, Shanghai Punan Hospital, Shanghai, China; 2 Department of Neurology, Renji Hospital, Shanghai Jiao Tong University School of Medicine, Shanghai, China; 3 Department of Neurorehabilitation, Shanghai First Rehabilitation Hospital, Shanghai, China

**Keywords:** Alzheimer’s disease, Epstein–Barr virus, intraventricular hemorrhage, lymphovenous anastomosis, seizures, tissue-based assay

## Abstract

Lymphovenous anastomosis (LVA) is emerging as a potential surgical intervention to ameliorate cervical lymphatic outflow and enhance glymphatic clearance in Alzheimer’s disease (AD). However, the spectrum of neurological sequelae associated with this procedure remains poorly characterized. We report the case of a 67-years-old male with amyloid PET–confirmed AD who underwent bilateral deep cervical LVA. Twenty-three days postoperatively, he presented with high-grade fever and altered consciousness. Head CT revealed acute hemorrhage in the posterior horn of the left lateral ventricle (∼2 mL). Cerebrospinal fluid (CSF) analysis demonstrated lymphocytic pleocytosis and significantly elevated protein levels; the fluid was uniformly bloody, confirming intraventricular hemorrhage. Plasma metagenomic next-generation sequencing (mNGS) identified Epstein–Barr virus (EBV), with serology supporting reactivation. Following antiviral and empirical antibiotic therapy, the patient’s condition stabilized, and the hemorrhage resolved. Four months postoperatively, he developed new-onset generalized seizures. Despite negative results from a conventional autoimmune encephalitis antibody panel in both serum and CSF, a tissue-based assay (TBA) proved positive in both samples. Seizures were successfully controlled with levetiracetam. This case suggests a potential association between invasive lymphatic procedures and a hemorrhage–infection–immune cascade in highly vulnerable AD patients with preexisting metabolic and neurodegenerative risk factors.

## Background

Alzheimer’s disease (AD) is the leading cause of dementia and is characterized by amyloid-β deposition and neurofibrillary tangles (Jack et al., 2018). Although anti-amyloid therapies have shown disease-modifying potential (van Dyck et al., 2023; Sims et al., 2023), their use is limited by safety concerns and cost, and complementary approaches aimed at enhancing brain waste clearance remain of interest. Increasing evidence supports a role for meningeal lymphatic vessels and glymphatic transport in clearing metabolites such as amyloid-β and tau from the brain to deep cervical lymph nodes (Iliff et al., 2012; Nedergaard and Goldman, 2020). On this basis, deep cervical lymphovenous anastomosis (LVA) has been proposed to improve lymphatic drainage and reduce outflow resistance (Xie et al., 2013). Although preliminary clinical explorations suggest potential cognitive benefits for AD patients (Li et al., 2024), the safety profile of this invasive intervention has not been fully characterized. Furthermore, the inherent blood–brain barrier (BBB) dysfunction and cerebral amyloid angiopathy (CAA) often seen in AD may significantly increase the perioperative risks of hemorrhage and infection dissemination (Chen et al., 2025; Zhang et al., 2025). We describe an AD patient who developed intraventricular hemorrhage, suspected EBV reactivation, and TBA-positive epilepsy of presumed autoimmune etiology following bilateral deep cervical LVA, and discuss the potential mechanisms and clinical implications.

## Case presentation

### Patient information and medical history

**FIGURE 1:**
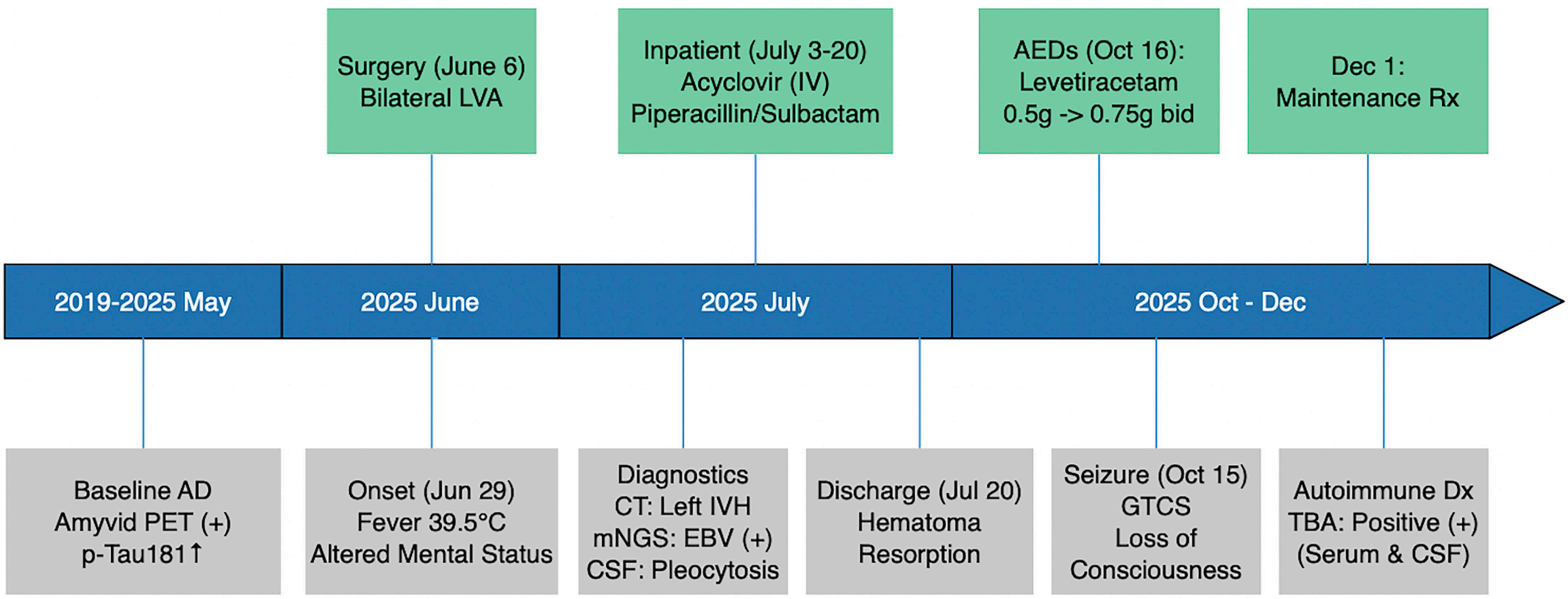
Overview of the patient’s clinical course, key diagnostic and therapeutic events, and outcomes. AD, Alzheimer’s disease; AEDs, antiepileptic drugs; bid, twice daily; CSF, cerebrospinal fluid; CT, computed tomography; EBV, Epstein–Barr virus; GTCS, generalized tonic–clonic seizure; IV, intravenous; IVH, intraventricular hemorrhage; LVA, lymphovenous anastomosis; mNGS, metagenomic next-generation sequencing; POD, postoperative day; Rx, treatment; TBA, tissue-based assay; Tmax, maximum body temperature.

A 67-years-old man (retired teacher) was admitted on 3 July 2025 with fever and slowed responsiveness for 5 days. He had type 2 diabetes mellitus for 10 years (treated with metformin) and AD diagnosed 6 years earlier (Figure 1).

**FIGURE 2:**
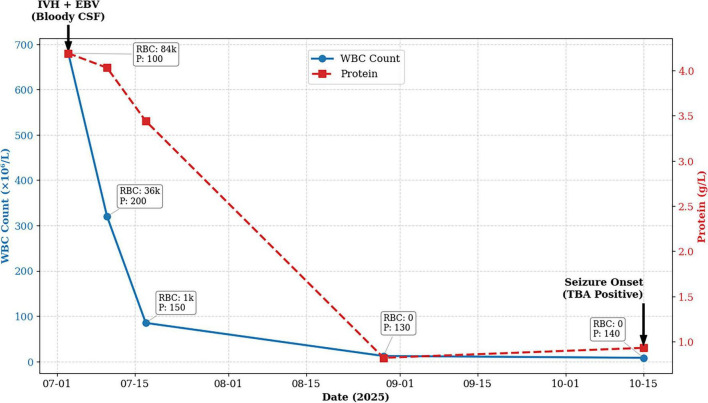
Temporal changes in cerebrospinal fluid parameters. RBC, red blood cell count (10^6^/L); P, cerebrospinal fluid opening pressure (mmH_2_O).

Progressive memory impairment began in 2019. Amyloid positron emission tomography with 18F-florbetapir (Amyvid) on 17 November 2021 demonstrated widespread cortical amyloid positivity involving the frontal, parietal, temporal, and occipital cortices, consistent with AD (Figure 2A). Despite treatment with lecanemab, memantine, and donepezil, his cognition continued to decline. Blood biomarkers on 27 May 2025 showed elevated p-Tau181 (76.04 pg/mL, reference < 20 pg/mL), low Aβ1–42 (441.86 pg/mL, reference > 600 pg/mL), and a reduced Aβ42/40 ratio.

### Surgical procedure and postoperative course

Following comprehensive preoperative evaluation to rule out surgical and anesthetic contraindications, the patient underwent bilateral deep cervical LVA under general anesthesia on 6 June 2025. As the surgery was performed at an outside institution prior to admission, obtaining further granular surgical details remains challenging. However, the available records explicitly confirm that the surgical procedure was uneventful, with no intraoperative complications such as direct vascular injury or hemodynamic instability. Postoperative management included routine intravenous fluids, anti-edema therapy, and hemostatic agents. Negative pressure drains were removed on postoperative day 3. His initial recovery was unremarkable, and he was discharged on 9 June 2025 without immediate complications or obvious cognitive changes.

### Clinical presentation

Twenty-three days postoperatively (29 June 2025), the patient abruptly developed sudden high fever (maximum 39.5°C) with chills, reduced speech, and impaired responsiveness. He was transferred to our department on 3 July 2025.

On admission and over the first 24 h, the patient remained hemodynamically stable without the need for vasopressors. Temperature 37.0°C–37.5°C, heart rate 75–85 beats/min, respiratory rate 18–20 breaths/min, blood pressure 106–125/66–80 mmHg. The quick Sequential Organ Failure Assessment (qSOFA) score was 1. Neurological examination revealed nuchal rigidity. Muscle tone was increased in all limbs. Deep tendon reflexes were symmetrically brisk, with a positive Babinski sign on the left.

### Investigations

Preoperative brain MRI (2 June 2025) showed periventricular and deep white matter hyperintensities on fluid-attenuated inversion recovery (FLAIR), consistent with Fazekas grade 2. Susceptibility-weighted imaging (SWI) showed no definite cerebral microbleeds (Figure 2B).

On admission (3 July 2025), head CT demonstrated a hyperdense lesion in the posterior horn of the left lateral ventricle (approximately 55–60 HU), consistent with acute intraventricular hemorrhage of approximately 2 mL (Figure 2C). Multiple lacunar hypodensities were present, mainly in the bilateral basal ganglia and corona radiata, with diffuse white matter hypodensity, widened sulci and fissures, and mild ventriculomegaly, consistent with cerebral atrophy. Follow-up head CT on 29 August 2025 showed absorption of the hemorrhage (Figure 2D).

Head and neck computed tomography angiography (CTA) on 4 July 2025 showed atherosclerotic plaques in the bilateral internal carotid and vertebral arteries with mild luminal stenosis; no intracranial aneurysm or arteriovenous malformation was detected.

Cerebrospinal fluid examination on 3 July 2025 showed uniformly bloody CSF across three consecutive tubes without appreciable clearing. After centrifugation, the supernatant was yellow, which was more consistent with true hemorrhage rather than a traumatic tap. Opening pressure was 100 mmH2O. Pandy test was positive. White blood cell count was 680 × 10^6^/L (lymphocyte predominant), red blood cell count was 84,000 × 10^6^/L, protein was 4.19 g/L, glucose was 4.2 mmol/L, and chloride was 118 mmol/L.

Plasma mNGS on 5 July 2025 detected Epstein–Barr virus (human gammaherpesvirus 4; 8 sequence reads, relative abundance 95%). Relative abundance refers to the proportion of EBV reads among all microbial reads. CSF mNGS was non-diagnostic because of severe hemolysis. CSF bacterial and fungal cultures and *Mycobacterium tuberculosis* PCR were negative. EBV serology (VCA-IgM+, VCA-IgG+, EA-IgG+, EBNA-IgG+) was suggestive of EBV reactivation, as the concurrent presence of VCA-IgM and EA-IgG indicates active viral replication rather than merely past dormant infection.

On admission, blood tests showed leukocytosis (11.2 × 10^9^/L) with neutrophilia (78%), hemoglobin 128 g/L, platelets 186 × 10^9^/L, D-dimer 5.79 mg/L, HbA1c 7.0%, and C-reactive protein 45.6 mg/L. Three separate sets of blood cultures obtained during the hospitalization were all negative. Lymphocyte subset analysis showed a mildly increased proportion of CD20+ B cells (14.91%, reference 7.44%–13.66%) and an increased proportion of circulating plasma cells (12.79%, reference 0.42%–2.82%). Cytokines were otherwise within the normal range.

A conventional (commercial) autoimmune encephalitis antibody panel (cell-based assay) was negative in both serum and CSF, including NMDAR, AMPAR1/2, LGI1, CASPR2, GABABR, DPPX, IgLON5, GABAAR (α1/β3), GlyRα1, mGluR1/5, D2R, neurexin-3α, GAD65, KLHL11, and ganglionic acetylcholine receptor (gAChR) antibodies.

Video EEG on 8 July 2025 showed a basic posterior rhythm of 8–9 Hz with poor modulation and frequent paroxysmal high-amplitude theta activity (4–6 Hz) with occasional delta activity (2–3 Hz) during wakefulness and sleep, without definite epileptiform discharges.

After seizure recurrence in October 2025, repeat EEG showed diffuse background slowing without captured epileptiform discharges. TBA on 16 October 2025 was positive in both serum and CSF; representative CSF staining is shown in Figures 2E, F.

### Treatment and outcome

**FIGURE 3:**
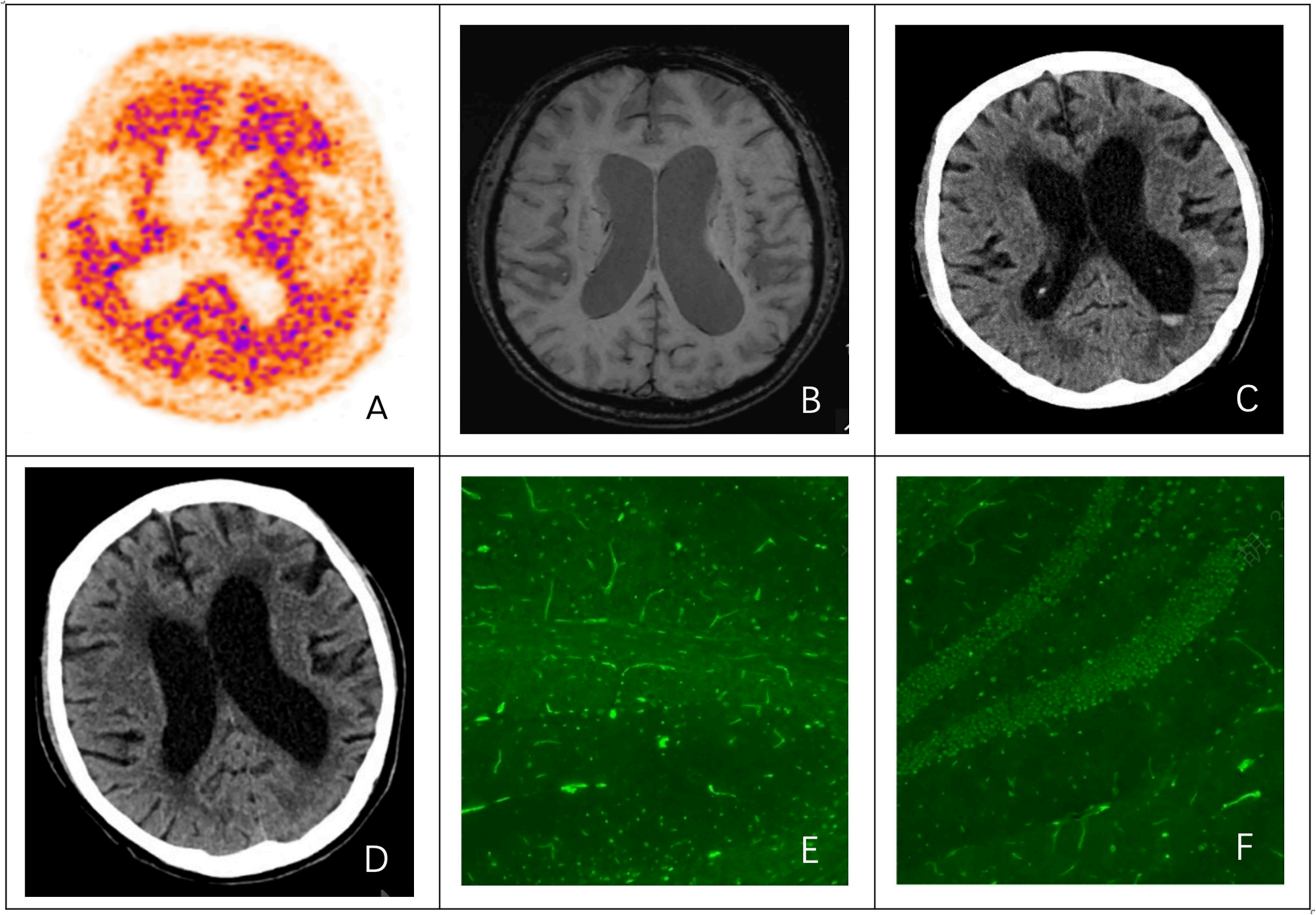
Neuroimaging findings and tissue-based assay (TBA) results. **(A)** 18F-amyloid PET showed widespread cortical tracer retention. **(B)** Susceptibility-weighted imaging (SWI) showed no definite cerebral microbleeds; background cerebral atrophy with mild ventriculomegaly was noted. **(C,D)** Head CT on 3 July 2025 showed a hyperdense lesion in the posterior horn of the left lateral ventricle; follow-up CT on 29 August 2025 showed absorption of the hemorrhage. **(E,F)** On 16 October 2025, TBA using mouse brain sections (including cerebellum) as substrates showed positive nuclear staining with the patient’s CSF (green fluorescence); serum showed a similar pattern (not shown).

He received intravenous acyclovir (0.5 g every 8 h) and empirical antibacterial therapy (ceftriaxone 2 g once daily, later changed to piperacillin–sulbactam 4.5 g every 8 h), along with supportive management including glycemic control. Serial lumbar punctures showed gradual improvement (Figure 3). On 10 July 2025 (second lumbar puncture), opening pressure was 200 mmH2O, protein 4.03 g/L, white blood cell count 320 × 10^6^/L, and red blood cell count 36,000 × 10^6^/L. On 17 July 2025 (third lumbar puncture), cerebrospinal fluid became clear yellow, opening pressure was 150 mmH2O, protein 3.44 g/L, white blood cell count 85 × 10^6^/L, and red blood cell count 1,000 × 10^6^/L. He became afebrile with improved mental status and was discharged on 20 July 2025. Prior to discharge, immune profiling on 18 July 2025 revealed persistent immune activation, characterized by elevated IL-8 (63.86 pg/mL), mildly elevated IL-6 (8.5 pg/mL), an increased CD19+ B-cell proportion (18.5%), and a reduced CD4+/CD8+ ratio (0.9). Follow-up head CT on 29 August 2025 showed complete resolution of the intraventricular hemorrhage; lumbar puncture showed clear cerebrospinal fluid with opening pressure 130 mmH2O, protein 0.82 g/L, white blood cell count 12 × 10^6^/L, and no red blood cells.

On 15 October 2025, he developed recurrent generalized tonic–clonic seizures (three episodes within 1 week). These episodes were characterized by sudden loss of consciousness and four-limb convulsions, lasting for approximately 2 min. Following the initial administration of antiepileptic medication, occasional focal limb twitching was still observed. The conventional autoimmune encephalitis antibody panel remained negative. However, TBA was positive in both serum and CSF. He was diagnosed with new-onset epilepsy with suspected autoimmune etiology (TBA positive; antibody panel negative) and was treated with levetiracetam (0.5 g twice daily, titrated to 0.75 g twice daily), with good seizure control. At follow-up on 1 December 2025, he had no further seizures, and IL-8 normalized (22.23 pg/mL).

## Discussion

This case demonstrates a rare triad of intraventricular hemorrhage (IVH), suspected EBV reactivation, and TBA-positive autoimmune epilepsy following bilateral deep cervical LVA in an AD patient. These findings underscore the potential risks of this novel surgical intervention.

The IVH occurred 23 days postoperatively, coinciding with EBV-induced fever. Rather than a direct causal consequence of LVA, this complication likely resulted from a “multiple-hit” process in a highly vulnerable patient. Several interacting mechanisms may explain this hemorrhage: (1) Baseline vascular fragility: the patient had a 10-years history of type 2 diabetes mellitus, which contributes to endothelial dysfunction and microvascular compromise. While cerebral amyloid angiopathy (CAA) is a common comorbidity in AD that increases vascular fragility (Greenberg et al., 2020; Jäkel et al., 2022), it is less likely the primary cause here. This is supported by the absence of definitive microbleeds on preoperative SWI and the ventricular, rather than typically lobar, location of the hemorrhage (Charidimou et al., 2012). Nevertheless, the inherent blood-brain barrier (BBB) dysfunction associated with long-standing AD remains a critical factor. (2) Surgical and hemodynamic triggers: LVA alters cervical lymphatic drainage, potentially causing fluctuations in intracranial venous pressure and cerebrospinal fluid dynamics, leading to compromised tight junctions and increased BBB permeability. Such disruption exacerbates vascular fragility and simultaneously facilitates peripheral pathogen entry into the central nervous system. (3) Infection and coagulopathy: EBV infection can induce endothelial injury and coagulopathy. Admission D-dimer elevation reflected activation of the coagulation-fibrinolysis system, with systemic inflammation further compounding vascular fragility. Notably, EBV encephalitis itself can present with hemorrhagic lesions, albeit rarely (Tselis, 2014). The simultaneous onset of fever and hemorrhage, alongside inflammatory CSF changes, suggests that hemorrhagic encephalitis cannot be definitively ruled out.

The high relative abundance of EBV sequences in plasma mNGS and positive serology (VCA-IgM and EA-IgG) confirmed EBV reactivation. This reactivation was likely triggered by a combination of surgical stress, AD-related immune dysfunction, and advanced age (Heneka et al., 2015). The mechanisms underlying EBV-associated neurological complications include direct viral invasion of the CNS, immune-mediated injury, and vasculitis (Tselis, 2014).

Approximately 3 months after the resolution of EBV infection and IVH, the patient developed seizures. The positive TBA and negative conventional autoimmune encephalitis antibody panel strongly suggest post-infectious autoimmune encephalitis. TBA is a critical tool for detecting neuronal autoantibodies (Masi et al., 2021). Previous studies indicate that 15%–20% of autoimmune encephalitis cases are TBA-positive but cell-based assay (CBA)-negative, suggesting the presence of autoantibodies against unidentified target antigens (Abboud et al., 2021). Mechanisms by which viral infections trigger autoimmune encephalitis include molecular mimicry, epitope spreading, and bystander activation (Dalmau and Graus, 2018; Graus et al., 2016). Thomas and Olsson (2023) systematically reviewed evidence of EBV triggering neuroimmune attacks via molecular mimicry, noting that cross-reactive T-cell receptors can simultaneously recognize viral and self-antigens. Similarly, Armangue et al. (2018) found that approximately 20% of patients with anti-NMDAR encephalitis have a history of preceding herpesvirus infection. The clinical course in our patient aligns with the classic paradigm of “viral infection → BBB disruption → neuronal antigen exposure → autoantibody production → autoimmune epilepsy.” The excellent response to levetiracetam further supports this diagnosis.

To mitigate these risks in future AD patients undergoing deep cervical LVA, we propose a structured perioperative framework. Given the current lack of large-scale evidence, this procedure should be approached with caution. Comprehensive preoperative screening is the critical first step, including high-resolution SWI to rule out cerebral microbleeds, coagulation profiling, and evaluation of latent viral infections (e.g., EBV serology). During the perioperative phase, management should focus on minimizing surgical stress and strictly controlling blood pressure to protect the vulnerable BBB. Furthermore, postoperative vigilance must extend well beyond the immediate recovery period. Clinicians should monitor for signs of infection, hemorrhage, and neuropsychiatric changes. As demonstrated by the approximately 3-months window from acute infection to autoimmune damage in this case, long-term follow-up is imperative. Particularly in patients presenting with new-onset seizures who are negative on conventional antibody panels but have a high clinical suspicion of an autoimmune etiology, TBA serves as an invaluable diagnostic tool.

## Data Availability

The raw data supporting the conclusions of this article will be made available by the authors, without undue reservation.
